# A systematic review and knowledge mapping on ICT-based remote and automatic COVID-19 patient monitoring and care

**DOI:** 10.1186/s12913-023-10047-z

**Published:** 2023-09-30

**Authors:** Ayan Chatterjee, Andreas Prinz, Michael A. Riegler, Jishnu Das

**Affiliations:** 1 Department of Information and Communication Technology, Centre for e-Health, University of Agder, Grimstad, Norway; 2 Department of Holistic Systems, Simula Metropolitan Center for Digital Engineering, Oslo, Norway; 3 Department of Information Systems, Centre for e-Health, University of Agder, Kristiansand, Norway

**Keywords:** Automatic monitoring, Remote monitoring, COVID-19, e-Health, Information and communication technologies, Artificial intelligence, Internet-of-things, Challenges, Semantic Ontology

## Abstract

**Background:**

e-Health has played a crucial role during the COVID-19 pandemic in primary health care. e-Health is the cost-effective and secure use of Information and Communication Technologies (ICTs) to support health and health-related fields. Various stakeholders worldwide use ICTs, including individuals, non-profit organizations, health practitioners, and governments. As a result of the COVID-19 pandemic, ICT has improved the quality of healthcare, the exchange of information, training of healthcare professionals and patients, and facilitated the relationship between patients and healthcare providers. This study systematically reviews the literature on ICT-based automatic and remote monitoring methods, as well as different ICT techniques used in the care of COVID-19-infected patients.

**Objective:**

The purpose of this systematic literature review is to identify the e-Health methods, associated ICTs, method implementation strategies, information collection techniques, advantages, and disadvantages of remote and automatic patient monitoring and care in COVID-19 pandemic.

**Methods:**

The search included primary studies that were published between January 2020 and June 2022 in scientific and electronic databases, such as EBSCOhost, Scopus, ACM, Nature, SpringerLink, IEEE Xplore, MEDLINE, Google Scholar, JMIR, Web of Science, Science Direct, and PubMed. In this review, the findings from the included publications are presented and elaborated according to the identified research questions. Evidence-based systematic reviews and meta-analyses were conducted using the Preferred Reporting Items for Systematic Reviews and Meta-Analyses (PRISMA) framework. Additionally, we improved the review process using the Rayyan tool and the Scale for the Assessment of Narrative Review Articles (SANRA). Among the eligibility criteria were methodological rigor, conceptual clarity, and useful implementation of ICTs in e-Health for remote and automatic monitoring of COVID-19 patients.

**Results:**

Our initial search identified 664 potential studies; 102 were assessed for eligibility in the pre-final stage and 65 articles were used in the final review with the inclusion and exclusion criteria. The review identified the following eHealth methods—Telemedicine, Mobile Health (mHealth), and Telehealth. The associated ICTs are Wearable Body Sensors, Artificial Intelligence (AI) algorithms, Internet-of-Things, or Internet-of-Medical-Things (IoT or IoMT), Biometric Monitoring Technologies (BioMeTs), and Bluetooth-enabled (BLE) home health monitoring devices. Spatial or positional data, personal and individual health, and wellness data, including vital signs, symptoms, biomedical images and signals, and lifestyle data are examples of information that is managed by ICTs. Different AI and IoT methods have opened new possibilities for automatic and remote patient monitoring with associated advantages and weaknesses. Our findings were represented in a structured manner using a semantic knowledge graph (e.g., ontology model).

**Conclusions:**

Various e-Health methods, related remote monitoring technologies, different approaches, information categories, the adoption of ICT tools for an automatic remote patient monitoring (RPM), advantages and limitations of RMTs in the COVID-19 case are discussed in this review. The use of e-Health during the COVID-19 pandemic illustrates the constraints and possibilities of using ICTs. ICTs are not merely an external tool to achieve definite remote and automatic health monitoring goals; instead, they are embedded in contexts. Therefore, the importance of the mutual design process between ICT and society during the global health crisis has been observed from a social informatics perspective. A global health crisis can be observed as an information crisis (e.g., insufficient information, unreliable information, and inaccessible information); however, this review shows the influence of ICTs on COVID-19 patients' health monitoring and related information collection techniques.

**Supplementary Information:**

The online version contains supplementary material available at 10.1186/s12913-023-10047-z.

## Key contributions to this review literature

• We systematically review different e-Health methods applicable to remote COVID-19 patient monitoring.

• We systematically review different data collection methods and data types collected by remote monitoring technologies in remote COVID-19 patient monitoring.

• We systematically review different approaches to design and develop an effective RPM system for COVID-19 patient monitoring case,

• We systematically review different ICT techniques to automate the COVID-19 patient monitoring.

• We systematically review benefits and shortcomings of RMTs in RPM systems for the COVID-19 patient monitoring.

• Semantic mapping and representation of obtained knowledge as an ontology model.

**Supplementary Information:**

The online version contains supplementary material available at 10.1186/s12913-023-10047-z.

## Introduction

### Overview

The COVID-19 pandemic has been a catalyst for developing and adopting widespread remote health monitoring technologies [1–4]. Indicating how these technologies were implemented in the early stages of this pandemic is essential in identifying barriers to their application and adoption, especially among the vulnerable populations [1–4]. The COVID-19 pandemic has pushed telehealth into the healthcare mainstream. One area of ​​telehealth that has seen particularly significant progress over the past two years is Remote Patient Monitoring (RPM). RPM benefits healthcare providers during the pandemic by facilitating hospitals that are already congested and allowing patients to be monitored and treated at home. Additionally, RPM reduces a patient’s time in situations where the COVID-19 virus potentially can spread [4]. In addition to the pandemic, RPM can keep patients away from high-cost environments in hospitals and at home, improving the patient experience while reducing costs [4]. A report based on data before the COVID-19 pandemic suggests that routine monitoring of hospitalized patients with continuous vital sign monitoring is associated with lower mortality [3]. Thus, with the advancement of ICTs, automatic and remote COVID-19 patient monitoring can play an increasingly significant role in health care to improve patient mortality by providing timely observation and care.

Societal structures, e-Health, and organizational processes are primarily influenced by ICT-driven information networks in today's global networked society. ICT, broadly defined as the internet, platforms, networks, telephony, applications, databases, and the underlying infrastructure, is a core element of the existing social order and e-Health, especially during the global COVID-19 pandemic. The importance of ICT extends beyond detecting, tracking, monitoring, managing, treating, and understanding epidemics. ICTs are our most effective chance to maintain social order and healthcare during the pandemic. During the pandemic, IoT technology has been used to monitor the health status of patients through wireless wearable sensors in various scenarios and diseases, such as non-communicable diseases and infectious diseases. Combining IoT-related technologies with early warning indicators commonly used in hospital wards can significantly improve healthcare delivery.

### Motivation

People with chronic medical conditions visit hospitals and clinics more often and are at higher risk for complications from COVID-19 [4]. As of June 2020, an estimated 41% of U.S. adults had delayed or avoided medical care, and people with chronic medical conditions were more likely to stay away from a hospital or clinic [4]. Even with state-of-the-art technologies, monitoring and managing patients who may be infected with COVID-19 remains a significant challenge. The disease can deteriorate rapidly in infected patients and continuous monitoring is usually required. The essential issue of COVID-19 is timely diagnosis and breaking the chain of transmission by isolating vulnerable population groups and patients. Thus, established technologies, operational infrastructures, and nursing resources should be used to develop an RPM plan for the care of COVID-19 patients. RPM has made considerable strides in collecting chronic patient data remotely [4]. An e-Health approach to remote monitoring of COVID-19 infected patients has many benefits, including early detection, reduced risk of transmission, efficient resource allocation, continuous monitoring, patient convenience, and data collection for research purposes. These motivations make remote monitoring an important tool in managing the COVID-19 pandemic.

ICTs are well-positioned to reduce the potential spread of disease and prevent the healthcare system from being overloaded with at-home COVID-19 screening, diagnosis, and monitoring. ICTs in e-Health are used to facilitate RPM [1–6] by offering the patients an ability to record, track, govern, analyze, and view their automatically recorded and self-reported data [7, 8]. ICT tools emphasize prospective information flow from patients to health professionals. The specific types of information collected may vary depending on the Remote Monitoring Technologies (RMTs) and the healthcare provider's requirements. However, the goal is to gather comprehensive and accurate data to assess the patient's condition remotely and provide appropriate care. The RMTs are a special category of ICT and use Internet-of-things (IoT) devices (e.g., wearable, and non-wearable sensors) for data collection. The nature of collected personal and health data is essential in COVID-19 automatic and remote patient surveillance and decision making. Adequate sensor and non-sensor data combined with data analytics approaches with Artificial Intelligence (AI) algorithms may improve predictive capabilities and allow for timely monitoring and management of health issues. The additional context of wearables can enable providers to make more informed decisions about a patient's care plan or treatment [4].

Remote monitoring has revolutionized healthcare by connecting remote and hard-to-reach areas [3–6]. The IoT-based health system [9, 10] has the potential of continuous health state monitoring both indoors and outdoors. Especially in the COVID-19 pandemic, it is imperative to have an automatic remote monitoring system to assess patients remotely and control their spread in advance. AI algorithms are helpful in processing sensory observations and medical images for automatic vital health sign predictive modeling [9, 10]. Therefore, IoT and AI can be promising and enabling health technologies to automate RPM processes. The motivation reveals that an automatic RPM process can be invaluable in remote patient monitoring and care; however, a robust system design is critical [9, 10]. Considering this, designing and developing an effective RPM system can enhance patient care, improve health outcomes, increase accessibility, ensure continuity of care, reduce healthcare costs, and protect patient privacy.

Automating the monitoring of COVID-19 patients using ICTs can bring benefits such as efficiency, real-time monitoring, remote access, scalability, timely intervention, data analysis, resource optimization, and contribution to research and surveillance efforts [1–6, 9, 10]. These motivations can drive the adoption of automation technologies to improve patient care and effectively manage the COVID-19 pandemic. However, a successful collaboration with IT professionals, healthcare providers, and relevant stakeholders is important to guarantee successful functioning and deployment of the automated monitoring systems. Furthermore, by recognizing the strengths and weaknesses of RMTs, healthcare systems and providers can leverage their strengths while addressing the challenges to ensure the successful implementation and use of RPM systems.

### Aim of the study

This systematic literature review aims to identify the relevant methods and different information management techniques to achieve ICT-based automatic and remote patient monitoring in COVID-19 infected patients care. In this context, the following research questions (RQs) have been formulated:

- RQ1: Which e-Health methods are used for remote COVID-19 infected patient monitoring?
- RQ2: What type of information is collected by RMTs in remote COVID-19 patient monitoring?
- RQ3: What are different approaches to design and develop an effective RPM system?
- RQ4: How to use ICTs to automate the COVID-19 patient monitoring?
- RQ5: What are advantages and drawbacks of RMTs in RPM systems?

## Methods

We performed a systematic literature review to formulate a detailed synopsis of the current literature on the topic in a reproducible and transparent way. We accomplished our systematic literature review following Wendler's proposals [11] to search online scientific databases. Systematic reviews convey a scientific synthesis of proof. It uses systematic and transparent strategies to comprehend, select, and strictly assess related basic research and extract and explore facts from the research covered in the evaluation [12]. We used the PRISMA [13] (see Supplementary Material—1) and Rayyan [14] evidence-based framework for the systematic review and meta-analyses. Afterward, we used the Scale for the Assessment of Narrative Review Articles (SANRA) [15] to assess the articles in the final round.

### Search strategy

We identified relevant publications in the following electronic databases EBSCOhost, Scopus, ACM, Nature, SpringerLink, IEEE Xplore, MDPI, Google Scholar, JMIR, Web of Science, and PubMed. This study’s search strategy was created in collaboration with the library of the University of Agder (UiA) in Norway. We identified related search keywords using the MeSH terms, synonyms, keywords from relevant articles, and self-determined search terms. The tools, such as “EndNote (V. X9)”, “DOAJ”, “Sherpa/Romeo”, and “Microsoft Excel (MS Office 365 V. 16.x)” were used to effectively search, collect, and select related articles. We filtered our literature search with the search string: *(COVID-19 or COVID 19) AND (remote monitoring OR automatic monitoring) AND (Technologies) AND (health monitoring),* and the used filters to narrow down the search list were: *Abstract, Free full text, Case Reports, Classical Article, Clinical Conference, Clinical Study, Clinical Trial, Clinical Trial Protocol, Comparative Study, Evaluation Study, Meta-Analysis, Observational Study, Randomized Controlled Trial, Review, Systematic Review, Humans, and English*.

### Eligibility criteria

This study strictly focuses on research articles related to automatic and remote monitoring of COVID-19 infected patients. Therefore, we aimed to include qualitative, quantitative, and mixed studies that described COVID-19 patients’ remote and automatic health monitoring methods with ICTs and associated existing and emerging techniques. The eligibility criteria for article selection are described in Table 1.

**Table 1:** The Eligibility criteria for article selection

Attributes	Inclusion Criteria	Exclusion Criteria
Article type	Peer-reviewed, full-length articles written in English, Journal papers, conference papers or books	Editorial, commentary, short paper, viewpoint, early-stage research report, white papers, posters, and papers written in non-English
Access type	Open-access, and accessible from university library	Subscription / chargeable publications
Indexing	Articles indexed in Google Scholar, and registered in Norwegian Register for Scientific Journals, Series and Publishers (kanalregister.hkdir.no)	-
Publication time	Jan 2020 – June 2022	-
Study focus	Automatic and remote monitoring of COVID-19 infected patients	Articles focused on non-COVID-19 and chronic illnesses
Study design	Qualitative, quantitative, and mixed method studies focusing methodologies, data governance, challenges, models, reviews, cohort, and longitudinal studies, statistical or numerical analysis, data analytics, and vital sign monitoring	Biological concept, robotic automation, non-remote patient monitoring, vaccination, face-mask safety, COVID-19 screening, emotion recognition, networking approaches, general survey report, awareness studies, post COVID monitoring, social isolation, prevention control, sentiment analysis, and social media data analysis
Outcome of interest	e-Health methods, AI techniques, automatic RPM approaches, non-contact vital sign monitoring, unobtrusive monitoring technologies, patient’s health, and wellness information management	Studies reporting exclusively on data privacy, security protocols, data standardization, budget, early protective methods, ICU Design, Teleproctoring, Telecardiology, and Telepsychiatry

### Data extraction

Data was collected in a structured format from a list of selected full-text research articles. In collaboration with the library of UiA, two researchers (AC and JD) conducted the electronic database searches independently. Furthermore, AC and JD cross-checked and hand-searched a few articles from online scientific databases to avoid missed articles during the initial search method. AC and JD prepared independent Microsoft Excel files to extract the following important information for article screening after the initial search – Title, Author names, type of the article (RPM or Automatic RPM), Publication Year, Scientific Database, Keywords, Abstract, Keynote from the Conclusion, Access Type (Open Access or UiA Subscribed Access, or Restricted Access), Technology Used, Technology Focus (e.g., deep learning, machine learning, IoT and wearables, IoT and Non-wearables, ontology), Study Focus (methodologies, information management, technology, challenges, new ideation), and Monitoring Subject (COVID-19 infected patients or other types of patients, e.g., recovered). AC and JD then filtered out their common search articles and prepared a master search list. MR and AP helped AC and JD to narrow down the master search list further by manual screening.

### Quality scoring for final selection

We divided and distributed initially selected articles among the authors to complete the screening using the Rayyan collaboration and research tool. After the individual screening, results were reviewed by other authors to eliminate differences between reviewers. The eligible peer review articles were identified by the manual review, quality score, and manual assessment of reference lists of related articles. First, we checked the Title, Keywords, Abstract, and Conclusion of the articles. We then independently reviewed each article to see if it qualified for final inclusion. In the prefinal assessment, for data extraction, we maintained an Excel spreadsheet with the following fields—Title, Reference, Author, Nature of the paper (review, conceptual, methodology, survey, and implementation), Year, Country of research, Key terms, Keywords, Publication channel, Technology use, Peer-reviewed, Key findings (outcome, measures, processes, methods, theory, components, and results), Nature of assessment (qualitative, quantitative, or both), Type of monitoring, Key challenges, and Quality score based on SANRA. The quality of the included articles was assessed using the SANRA 0–2-point scale. We graded individual papers based on the six quality parameters defined in (Table 2).

**Table 2:** SANRA scaling, based on the quality parameters

• Explanation of the article’s importance for readership,
• Statement of specific aims or formulation of questions,
• Conference and journal papers to explain the overview of this study,
• Referencing,
• Scientific reasoning, and
• Appropriate data presentation

Individual quality parameters were further categorized on a 0–2–point scale. Finally, we calculated the mean score of the six quality parameters. The number of studies included in the pre-final assessment was 102, with a SANRA score greater than 1.8.

In the final assessment, 65 articles achieved a SANRA score greater than the cut-off of 1.9. Table 3 describes the research topics and types of scientific articles identified and selected in the final assessment. [2–4, 7–19], and [20] are studies relevant for study narration and formulation and are not a part of 65 papers selected through this systematic literature review.

**Table 3:** Research topics and count of articles with SANRA score > 1.9

Main Research topics^a^	Count (n): n > 0
COVID-19 + RPM + Design Methodology	3
COVID-19 + RPM + IoT	27
COVID-19 + RPM + AI	26
COVID-19 + RPM + Automation	24
COVID-19 + RPM + Challenges / Management / Program Modelling / Usefulness	18
COVID-19 + RPM + General ICT overview	3
COVID-19 + RPM + e-Health Methods	8

^a^We found overlapping research topics in several studies

### Knowledge representation

Our key findings for each RQ are presented in the form of ontology mapping (an object-oriented paradigm). Ontologies play a crucial role in knowledge representation and are essential for enabling intelligent systems to understand and work with complex, domain-specific information. They provide a foundation for building knowledge-based applications that can automate reasoning, decision-making, and information retrieval tasks. We designed and developed the ontology mapping in the Protégé (v. 5.x) open-source software and visualized the ontology using the OWLViz tool in Protégé [16, 17]. In the object-oriented representation, owl:Thing acts as a global parent class and the arrows define a hierarchical relationship (IS-A) between the concepts [18, 19].

## Results

### Selected articles and corresponding research contribution(s)

**Fig. 1:**
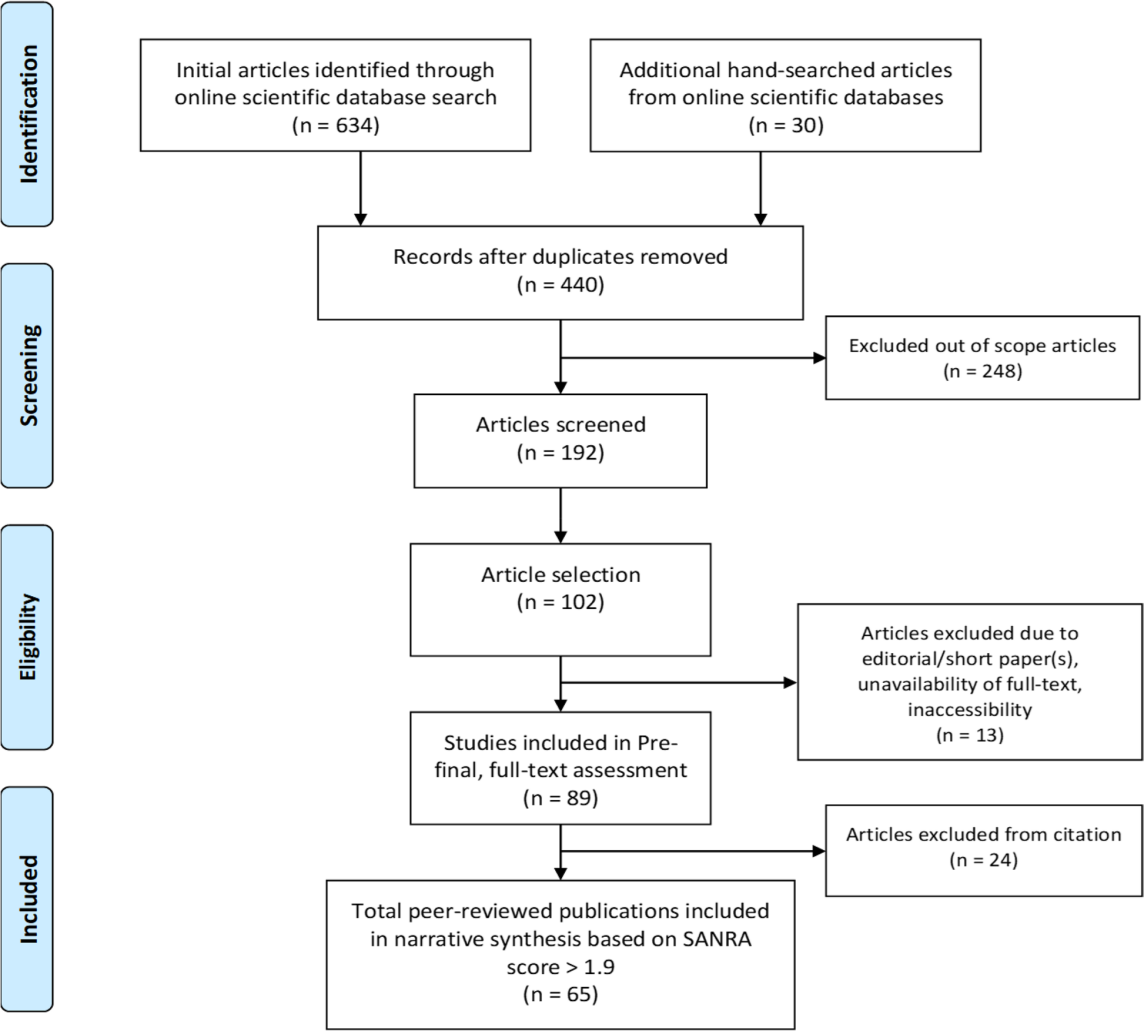
PRISMA flow diagram of the performed systematic literature review

**Fig. 2:**
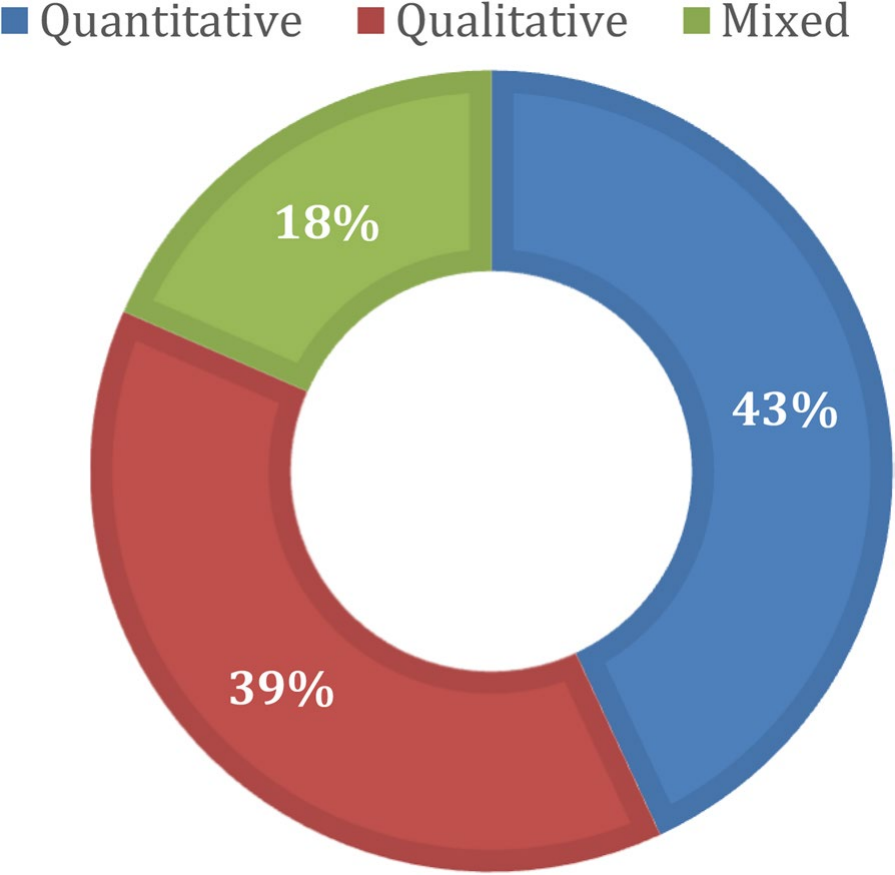
Percentage (%) of qualitative, quantitative, and mixed studies

The electronic search resulted in 664 articles, including 196 duplicates. In the pre-final stage, we selected 102 articles for full-text review after checking the abstract, conclusion, length of the paper, and the availability of the full text. In the final stage, we included peer-reviewed publications only, resulting in 65 peer-reviewed articles from different countries, such as the USA, China, Canada, UK, Portugal, Australia, India, Italy, Japan, Oman, Egypt, Saudi Arabia, Brazil, Bangladesh, Iran, Iraq, Turkey, Tunisia, Germany, and France. The articles are related to ICT-based remote and/or automatic COVID-19 infected patient monitoring, AI decision making models, e-Health methods, wearable IoTs, ontology-based IoT, information management, usefulness, and associated challenges. Most of the selected articles are indexed in PubMed (*n* = 52; 80%). The PRISMA flow diagram in Fig. 1 summarizes the entire process of article searching, filtering, and selection. Figure 2 illustrates the percentage (%) of qualitative, quantitative, and mixed studies.

Table 4 groups selected studies according to their focus and categories based on the key areas in ICT-based remote and automatic COVID-19 patient monitoring and care, comprehensive literature search with appropriate keywords and search terms related to each category to ensure comprehensive coverage, summarization of main outcomes, results, and conclusions of the study related to the specific research category, implications of the research for healthcare practice, policy, and future research directions. According to Table 4, the research focus can be classified into nine categories to understand the articles better to answer the RQs. In Table 4, we have cited sources of each category appropriately to provide an accurate and reliable summary.

**Table 4:** Included studies, their research category and key findings

Research focus	Key research categories	Studies^a^
Machine learning and deep learning-enabled remote monitoring of vital signs for COVID-19 patients	COVID-19 automatic diagnostic and predictive tools, determination of disease severity, and collection of remote health data through connected medical devices	[21–40]
IoT-based early-warning architecture for remote COVID-19 patient’s health monitoring	Smart IoMT wearables for monitoring patients’ health in different scenarios and medical conditions, geofencing, unobtrusive monitoring technologies, framework	[21, 25, 27–31, 35, 38, 39, 41–55]
Smart ontology based IoT in RPM	Ontology model, IoT framework, knowledge representation and reasoning approach	[56]
Deep learning techniques for computer vision-based COVID-19 patient monitoring in remote settings	COVID-19 automatic diagnostic and predictive tools based on biomedical image collection and processing	[23, 24, 26, 31, 34, 36, 37, 55–62]
Implementation of a multisite and interdisciplinary RPM program	Approaches, usefulness	[45, 63–66]
Observational study program	Statistical analysis on population data to understand the successful use of different ICT tools	[1, 5, 21–23, 28, 29, 36, 52, 55, 58–60, 62, 67–69]
Barriers to use RMTs	Identification of perceived benefits and barriers to use RMTs	[21, 28, 65, 70–74]
Importance of general ICT during COVID-19 pandemic	Roles, importance, societal benefits, and crisis	[6, 72, 73, 75–77]
e-Health-based remote COVID-19 monitoring, and management	Different e-Health methods	[43, 54, 65, 67, 71, 73, 78–81]

^a^We found overlapping research topics in several studies

Supplementary Table 1 (see Supplementary Material—2) describes selected publication types, e-Health methods (such as telehealth, telemedicine, and mHealth), used RMTs for required health data collection, and the collected data types. The data has been collected with different RMTs, such as smartphone applications, wearable devices (such as, smartwatches, fitness trackers, temperature patches), connected health devices (such as, pulse oximeters, glucose monitors, blood pressure monitors), and remote monitoring platforms. Moreover, Supplementary Table 1 represents monitoring of symptoms, medication adherence, and physiological parameters (or vital health signs, such as heart rate, blood pressure, respiratory rate, body temperature, glycemic response, oxygen saturation) from COVID-19 infected patients under remote care settings.

Supplementary Table 2 (see Supplementary Material—3) describes adopted approaches to designing and developing an RPM system. It is crucial to recognize that these approaches are not isolated from one another (i.e., mutually exclusive). Therefore, a combination of different approaches can be employed to create a comprehensive and effective remote patient monitoring system. The process of selecting an approach depends on the specific requirements, resources, and technological infrastructure in a particular healthcare setting. By following these approaches, the design and development of a RPM system can be enhanced to precisely meet the needs of patients and healthcare providers, facilitate effective remote patient monitoring, and finally improve the outcomes of patients and enhance healthcare delivery.

In medicine a *sign* is defined as an objective, observable health problem that another person can recognize; whereas a *symptom* (e.g., Pain, fever, fatigue, and difficulty breathing) is the manifestation of a medical condition that a patient is experiencing. Symptoms can be subjective (when they are experienced only by the patient) and objective (when they can be observed by a healthcare provider). They are not the same as diseases as they are simply indications that something is wrong with the body. A wide variety of factors, such as infections, injuries, and chronic medical conditions can cause them. The most critical medical signs are vital signs, such as heart rate, respiratory rate, body temperature, glycemic response, and blood pressure. Symptoms are chronic, relapsing, and remission. In this review, we identified diagnostic signs and chronic symptoms. Vital signs help to diagnose specific disease symptoms. According to the review, RPM can be perfectly integrated with telehealth when a specific health condition of a COVID-19 patient needs to be monitored. The ability to monitor certain aspects of a COVID-19 patient's health from home has become an increasingly popular telehealth option and can reduce patients' travel costs and infection risk.

Supplementary Table 3 (see Supplementary Material—4) describes different study designs, statistical analysis on population data or public datasets in automatic and/or remote COVID-19 patient monitoring systems, corresponding research outcomes, and AI model performances (if applicable). We have divided the study design into the following three categories: Random controlled study, empirical study, and mixed study (a combination of both). The random controlled studies primarily include a qualitative evaluation, whereas the empirical studies focus on a quantitative evaluation. The studies included in Supplementary Table 3 belong to the "Observational study program" category as mentioned in Table 4.

Supplementary Table 4 (see Supplementary Material—5) elaborates on studies related to the automatic RPM process. RMTs offer several advantages and drawbacks. Table 5 explains the benefits, and Table 6 presents the barriers to RMTs for COVID-19 patients.

**Table 5:** Benefits of using RMTs

Benefits	Studies^a^
Reduced burden of care	[6, 30, 43, 45, 49, 63, 64, 68, 70, 75, 78, 80]
Supports required populations	[6, 43, 44, 49, 63, 64, 68, 70, 76, 78, 80]
Easy remote health data collection, data analysis, and monitoring	[6, 21, 30, 41, 43–45, 49–51, 63, 64, 67, 68, 70, 75, 76, 78, 80, 81]
Improves user (or COVID-19 patient) experience	[6, 49, 50, 66, 68, 70, 75]
Improves remote care and mortality (or health outcomes)	[6, 21, 30, 43, 45, 49, 67, 68, 70, 76, 78, 80]
Technology-related benefits	[6, 21, 30, 41, 43–45, 49, 50, 63, 64, 67, 68, 70, 75, 76, 78, 80]
Cost effective virtual sign monitoring	[21, 41, 44, 51, 64, 70, 78, 80]
Easy to use	[1, 43, 70, 78, 80]

^a^We found overlapping research topics in several studies

**Table 6:** Barriers to use RMTs

Barriers	Studies^a^
Lack of technology experience	[21, 70–73]
Lack of implementation guidelines	[21, 24, 70–72]
Ethical concerns	[21, 28, 56, 70–72, 74, 76]
Problem with technology integration and/or technology changes	[5, 65, 70–74]
Quality care and quality information	[70–72, 74]
Communication dilemma	[65, 70, 72]
RPM model variation with patient triage, monitoring, and escalation	[70]
Staff workloads and budgets	[21, 70]
Insufficient information for decision making	[6, 24, 70–72]

^a^We found overlapping research topics in several studies

### Answering to RQs

The research findings captured from Table 4 to Table 6 helped to answer our identified RQs. This systematic literature review identified 12 studies contributing to answering RQ1 (see Supplementary Table 1); 24 studies contribute to our RQ2 (see Supplementary Table 1); 28 studies contribute to our RQ3 (see Supplementary Table 2); 19 studies contribute to RQ4 (see Supplementary Table 4); 28 studies contribute to RQ5 (see Table 5 and Table 6 (several studies contributed to more than one RQ).

**Fig. 3:**
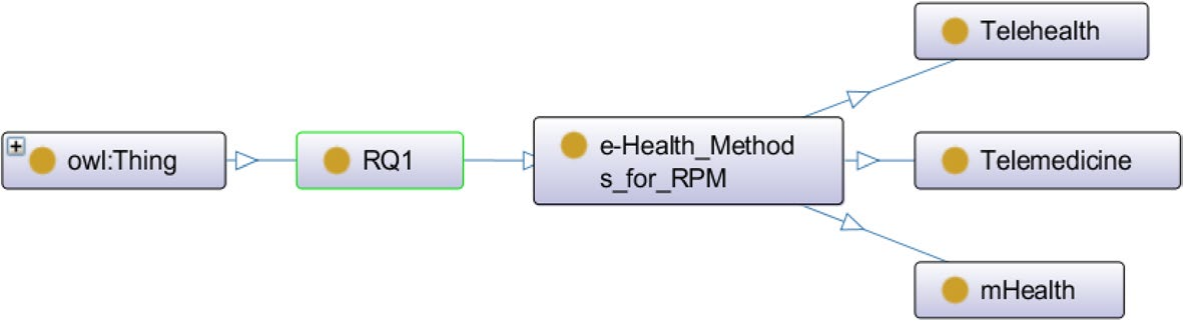
The semantic representation of eHealth methods for ICT-based remote COVID-19 patient monitoring and management

COVID-19 results in comorbidities (e.g., respiratory symptoms, cardiovascular symptoms, diabetic symptoms); thus, a continuous health monitoring is important [20]. A wide range of ICTs have been used to improve public health strategies, and the pandemic has increased the use of e-Health methods. To answer RQ1 we reviewed different e-Health methods and digital transformations as a part of remote health monitoring in COVID-19 patients. The identified e-Health methods for ICT-based remote COVID-19 patient screening, diagnosis, monitoring, planning, and consultation have been Telehealth, Telemedicine, and mHealth (see Fig. 3). The collection and assessment of data are essential to monitor, measure, assess, and diagnose COVID-19 risks associated with the care and monitoring of an infected patient (home quarantined or hospitalized).

**Fig. 4:**
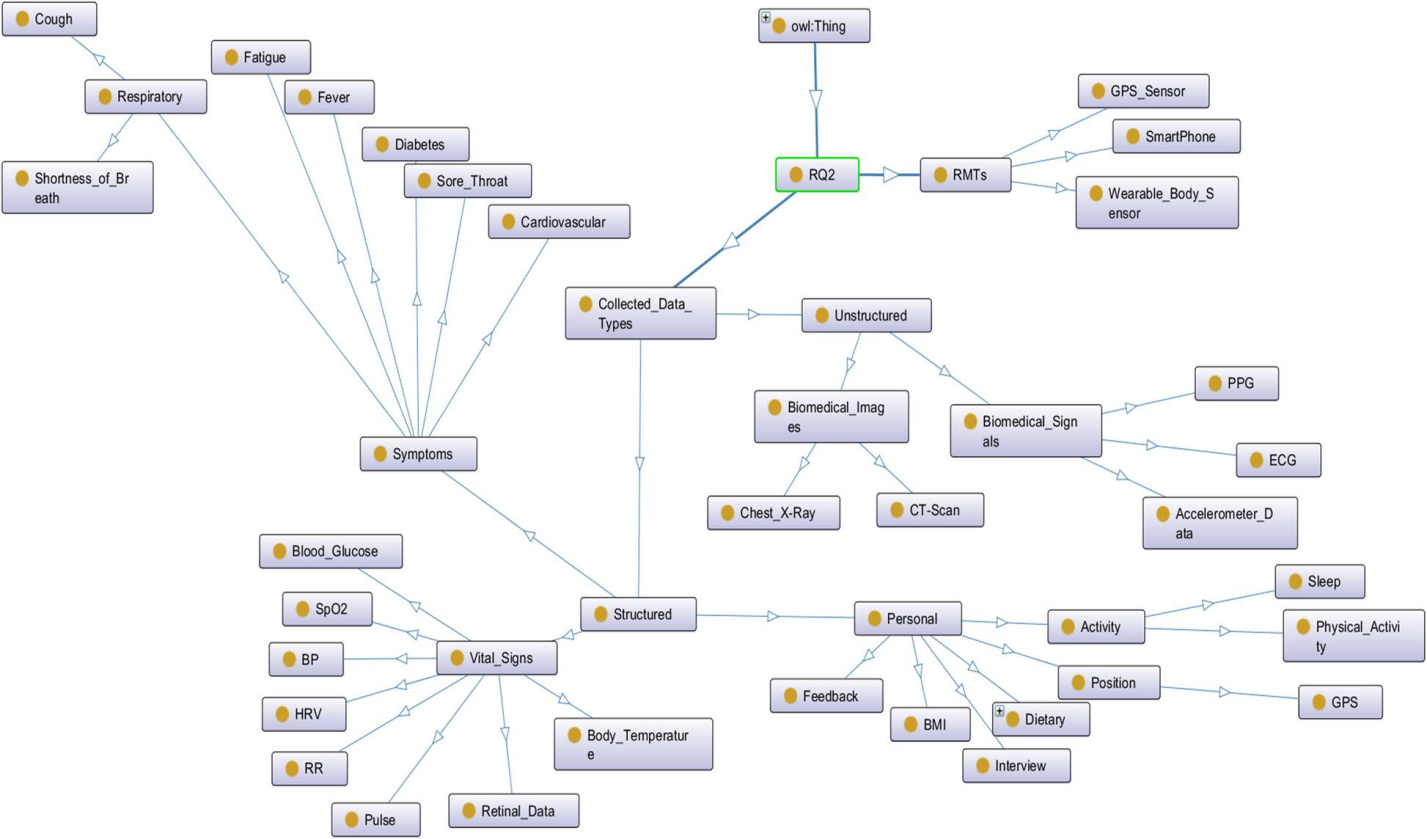
The semantic representation of processed data collected in RPM of COVID-19

For RQ2, we reviewed different data types and their collection process with RMTs for remote COVID-19 patient monitoring. Continuous assessment of health and wellness data is essential for automatic detection of COVID-19 disease and predicting the severity for better decision-making and readiness. Several e-Health methods have been deployed with a focus on assessing and managing data related to patient’s context, personal information, biomarkers, health, and wellness. Vital signs and symptoms associated with COVID-19 have been collected with smart wearable devices, smartphones, video consultation, online questionnaires, and feedback generations over on-premises or cloud platforms. Smartphone applications help to collect daily lifestyle data. Body temperature collected with smart biosensors is an early warning sign of infection and a symptom of fever. ECG, HRV, PPG, respiratory rate, oxygen saturation, and blood pressure collected with smart biosensors are important for cardiovascular symptoms and stress management. Retinal data and glycemic response are important for diabetes symptoms. Figure 4 gives a structural representation of the collected data.

**Fig. 5:**
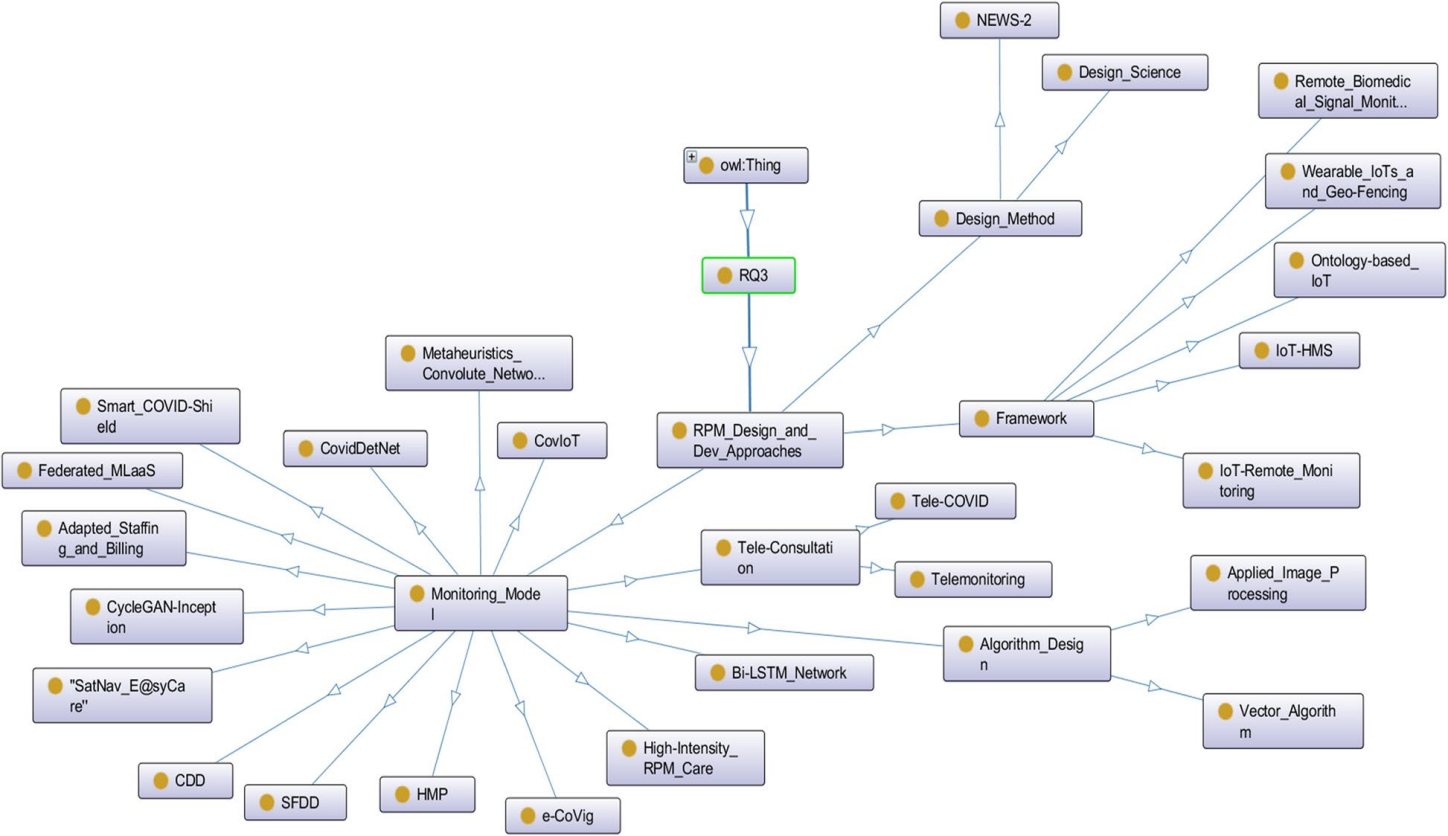
The semantic representation of RPM design and development approaches in COVID-19 patient monitoring

For RQ3 we identified different approaches to designing and developing RPM processes (see Fig. 5). We found IoT platforms to be a promising technology for rapid diagnosis, dynamic monitoring and tracking, and better treatment and control without spreading the COVID-19 virus to non-infected individuals. Solutions such as HMP, computerized analyzers, tele consultation models, IoT-HMS framework, IoT-Remote patient monitoring framework, and AI-based decision-making helped stakeholders, such as governments, experts, medical staff, and citizens to prevent and respond to remote COVID-19 patient monitoring, incidence management and care.

**Fig. 6:**
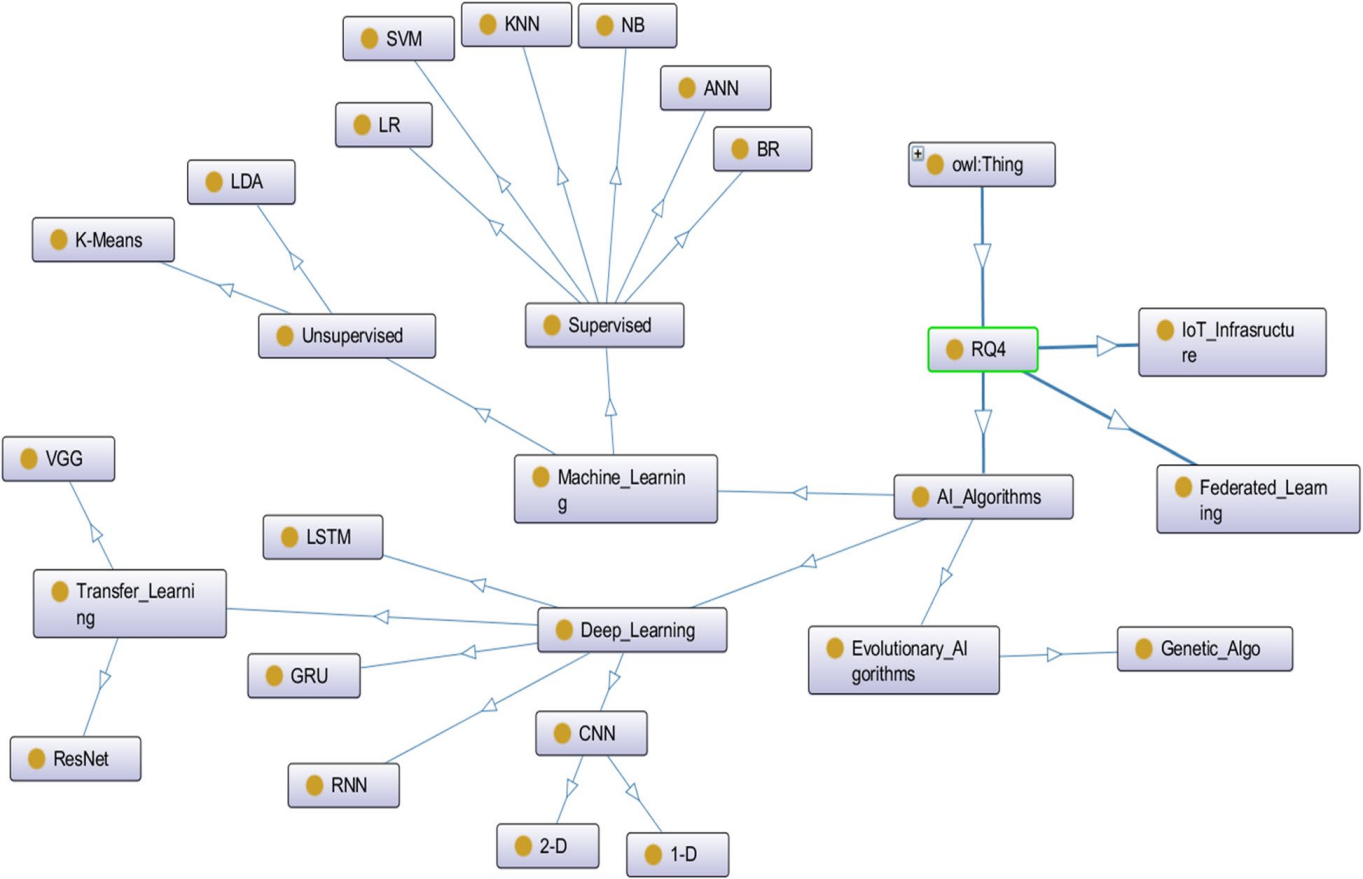
The semantic representation of ICT techniques used in automatic and remote COVID-19 patient monitoring

The success of the RPM program in COVID-19 patient monitoring has been dependent on the identification of the key characteristics, appropriate implementation of RMTs and ICT techniques. RQ4 reviewed ICT techniques (e.g., IoT, Deep Learning, Machine Learning, and Federated Learning) for an automatic and remote COVID-19 patient monitoring (see Fig. 6). The machine learning and deep learning techniques have been beneficial for analyzing cough, voice, and breathing acoustic, image (e.g., chest X-ray, CT-scan) and signal processing (e.g., time-series), image segmentation (e.g., U-Net), predictive modeling based on real-time symptom, vital signs and medical records, pattern recognition and clustering of symptoms against the following performance metrics – accuracy, precision, recall, F1-score, and area-under-curve. The proposed federated model [32] ensures accurate long-term AI-based decision-making using federated batch machine learning algorithms installed on the cloud infrastructure. The IoT technologies helped to design and develop simple, reliable, and low-cost microcontroller-based wireless monitoring system that can automatically record vital physiological data. In continuous and real-time patient monitoring, all the collected health and wellness data can be transferred to the backend server or cloud storage using an IoT communication and secure protocols for future decision-making and alert generation. The used deep learning algorithms have been LSTM, 1D-CNN, 2D-CNN, RNN, transfer learning (e.g., VGG-16, ResNet50), and GRU. The associated machine learning algorithms have been unsupervised (K-Means, LDA), and supervised (ANN, LR, SVM, BR, NB, KNN, and CART).

For RQ5 we identified associated benefits and drawbacks with the usage of RMTs in the RPM program for COVID-19 remote patient monitoring and care. In particular, the literature review has highlighted the requirements, opportunities, and drawbacks for RMTs in the RPM healthcare system.

## Discussion

### Principle findings

The COVID-19 pandemic has shown the importance of RPM and management to provide timely care [63, 64]. Automation in remote monitoring is a promising and challenging research task. RPM involves RMTs that allow healthcare services to track individuals [21, 41, 64, 70]. An automatic RPM system requires a cyclic process of data collection, data processing for health status monitoring, decision-making based on the processed data, recommendation generation, and action to be performed based on the care input by the patients and/or caregivers [6, 21, 41, 42, 64, 70]. Here, we discuss the findings related to identified research questions.

The advent of electronics for wearable sensors and emerging telemedicine tools helped to address remote COVID-19 patient monitoring, and management deficiencies [1, 5, 63, 64]. Telehealth offered multiple modes (e.g., telecare) and means (e.g., IoT devices) of interaction for COVID-19 patients and physicians. Telehealth attempted to meet the needs of COVID-19 patients by sensing disease progression and preventing life-threatening conditions [5, 30, 63, 67, 73]. In literature, the words "telemedicine" and "telehealth" are frequently used. The relationship between telehealth and telemedicine varies depending on the source and context; however, telemedicine is a subcategory of telehealth. Telehealth is a broader term that includes both clinical services and a broader range of remote healthcare services and activities. Telehealth includes telemedicine conversations, but it also includes remote patient monitoring, health education, administrative functions, and non-healthcare aspects that are telecommunicated through technology. Telemedicine is concerned with the utilization of telecommunication technology for remote healthcare services, such as virtual conversations between patients and providers that lead to diagnoses, treatments, and follow-up care. Telemedicine primarily apprehensions the transmission of clinical services over the internet.

Heart disease, diabetes, chronic obstructive pulmonary disease, and obesity are increased risk of severe illness from COVID-19. Therefore, continuous monitoring of vital physiological parameters and symptoms are essential for COVID-19 patients. Therefore, Supplementary Table 1 represents different types of data, purpose, and their nature (processed and/or raw) and their collection methods. It necessitates the transformation in patient tracking and caregiving through the integration of remote technologies. Recent advancements in ICT techniques, e-Health methods, and continuous remote monitoring have become essential in managing health symptoms and vital health signs of COVID-19 patients in the remote care settings [6, 72, 73, 75–77]. These technologies have changed the landscape of RPM and become more useful than ever. By leveraging exciting developments in personalization, digitization, health, and patient engagement, healthcare providers have improved health outcomes. In remote monitoring, necessary digital tools are required to collect health metrics and make the recorded data usable, reasonable, and applicable. Data exchange and recorded health and wellness data via secure messaging technologies with store-and-forward services had the lowest penetration rates in virtual COVID-19 disease management. The choice of telehealth communication services and interaction patterns in use cases, disease categories, and applications has played a key role in the success of telehealth, telemedicine, and mHealth management infrastructure and its impact on a better future healthcare system [21, 63, 64, 71].

This systematic literature review has discussed the contributions of ontology, IoT, and AI to propose complementary and multidisciplinary technologies to combat COVID-19, providing opportunities for more comprehensive research and accelerated knowledge acquisition and knowledge representation [21, 27, 45, 46, 56, 65, 68]. Supplementary Table 2 presents different RPM design approaches (e.g., design science), design framework, algorithm design, and various initiatives such as mobile apps, web platforms, and smart analytics for early detection and general COVID-19 health management. AI plays a crucial role in measuring, assessing, and diagnosing risk.

According to Supplementary Table 3 and Supplementary Table 4, AI has been a useful tool to detect and predict, at a large-scale, COVID-19 infected patients health conditions in a remote care setting. However, the exponentially rapid increase in the number of patients requires efficient, fast, and accurate predictions of the possible outcomes of COVID-19 infected patients for appropriate treatment using AI techniques. There is an urgent need to integrate AI technologies into wireless infrastructure (IoT/IoMT), real-time detection, and processing of end-user devices [25, 26, 31, 39, 53]. ICTs have become one of the essential components in developing connected smart IoMT devices. The data generated by IoMT devices is increasing rapidly due to the number of connected devices [31, 39]. The state of the COVID-19 outbreak has led to the need for IoMT, which can provide effective automated monitoring solutions. Therefore, analyzing IoMT data is extremely important. AI is gaining attention in automation applications based on big data generated by IoMT devices. AI and IoMT-driven telemedicine technology is a new-generation telehealth solution for remote and automated COVID-19 patient monitoring, which will redefine RPM solutions with AI and IoMT technology.

Remote monitoring of COVID-19 patients at home has raised trust issues related to RMT's ethical data collection; therefore, transparency and data protection must be guaranteed [6, 21, 41, 42, 63, 64, 70, 75, 78, 79]. Using ICT to monitor COVID-19 patients presents several ethical challenges [21, 70, 76]. One of the major concerns is the possibility that personal health information may be collected and processed in a manner that violates the privacy of patients. Information collected should be used solely for the purpose of providing medical care and should not be shared with third parties without the consent of the patient. When deploying ICT to monitor patients, there is also the risk of data breaches and cyberattacks. To ensure that patients' personal and medical information is protected, appropriate security measures must be in place. The inability of some patients to access technology or an internet connection may result in disparities in the quality of care. No matter what their technological capabilities are, all patients should have access to the same level of care. Moreover, information collected via remote monitoring may not be as accurate as information collected through in-person assessment. Therefore, the information collected should be reliable, and patients should receive appropriate follow-up care based on the information collected.

Chronic conditions have additional complexity and require long-term data to identify trends in COVID-19 patient conditions or measure the effectiveness of remote monitoring programs [6, 24, 70–72]. According to our review, ICT solutions such as wearables are closing the gap between clinical diagnostic devices and short-term fitness wearables by creating solutions that take clinical sensors, connect them, and use them for long-term data collection, and enhance the data availability for AI-assisted analytics. Combining continuous data with RPM and data analysis approaches (see Supplementary Table 2), AI can improve predictive capabilities for more timely intervention and management of remote patient monitoring. AI and sensor fusion technologies, including IoT/IoMT solutions, have shown promising results with enhanced potential for remote monitoring through ideal wearable solutions for long-term monitoring of COVID-19 patients- (see Supplementary Table 3 and Supplementary Table 4). The use of remote monitoring systems and associated technologies can complement face-to-face consultations and potentially increase COVID-19 patients' autonomy in treatment and disease management to decrease the no-show rate and the patients’ waiting times.

The use of wearable mobile devices as an early detection and real-time monitoring tool can enable remote monitoring to address unprecedented challenges. According to Tables 8 and 9, the RPM systems are of excellent help not only to COVID-19 patients but also to healthcare professionals. However, RPM still only works for some afflicted people, depending on their location and remote access capabilities. Additionally, physicians need to use extra effort to engage patients and motivate them to use RPM. Another disadvantage of RMP is that the device's accuracy needs to be proven [6, 21, 24, 70–72]. If the possibility of inaccuracy exists, the effectiveness of RPM remains uncertain.

## Challenges in rapid technology deployment

Healthcare services implementing RPM should anticipate the challenges associated with rapid technology deployment (see Table 6) and provide appropriate support to address these challenges, including strategies to support consumers' use of health information technology. Despite remarkable technological advances (see Table 5), extensive hospital and home remote monitoring services are not widely used by the healthcare system. Before healthcare systems can benefit from remote monitoring and automated patient management services, they must overcome the following hurdles – *a. selection of algorithms:* continuous monitoring can improve safety and reduce clinician workload. This approach should be combined with better algorithms to use automatic monitoring and clinical data to diagnose the cause of clinical deterioration and recommend treatment, *b. task management:* a service line must be established to monitor and manage patients with COVID-19. Generally, a group of physicians, such as emergency medical technicians or hospital physicians, execute a home monitoring program, develop protocols, admit, and monitor patients, and manage the quality of care. This service line should also have a research focus to understand better if, how, why, and in whom these therapies improve quality and value, *c. data privacy:* the synergy of IoT/IoMT, AI, and Federated Learning faces challenges related to privacy, latency, and lack of context. Emerging edge intelligence technologies may provide an opportunity to address such issues. AI algorithms should be used to make better and automatic predictions from limited public health data. Security and privacy methods can be used to protect sensitive health information, *d. limited knowledge:* there is evidence of reluctance to manage telehealth systems due to low technology adoption rates and a lack of support from public or private entities in developing technology to provide health services that meet people's needs, *e. limited funding:* insufficient hospital funding and a lack of patient attention pose challenges in hotspots. By providing care delivered remotely, we can focus on in-person care in the hospital or at home only for those who need it. It could enable systems to expand, protect lives and safeguard economic activity, *f. collaboration across vendors:* value maximization is required when healthcare systems combine and integrate multiple technologies such as monitoring, telemonitoring, chatbots, triage and scheduling, and *g. standards*: appropriate selection and enrollment of protocols that match patient risk and needs, with a menu of monitoring types and durations, required monitoring, and responses to patient deteriorating conditions or abnormal readings, *g. protocols* must address patient needs through therapies such as home health care services, pharmacy services, physical therapy, laboratory, and imaging testing, and telemedicine physician or clinic services, *h. the Role of AI*: Even with clinical evidence, RPM can improve efficiency without AI. With AI, we can improve efficiency by orders of magnitude. The AI-based RPM identifies patients potentially needing attention based on AI analysis of data. With the help of AI, RPM has the potential to be an essential part of the solution to soaring healthcare costs due to an aging population. AI-based RPM identifies patients who may need attention based on AI data analysis. Additionally, an AI decision support module can also be used alone to diagnose such COVID-19 patients. For example, AI can automatically change a patient's medication, direct a patient to a lab for additional testing, or advise a patient on diet and exercise, and *i. Population demography:* Most of the reported studies focus on adults and very limited studies focused on elderly population. A diversity in study is needed.

### Review

We have identified the following limitations in this literature review – a. the availability and quality of the relevant studies have been limited on this topic, b. the lack of related technical and empirical evidence-based studies on high-quality randomized controlled trials, c. studies scoped on this topic use various designs, methodologies, and outcome measures, leading to heterogeneity in the data which causes challenges to directly compare and combine study outcomes, hypothetically affecting the meta-analysis and generalizability of the findings, d. major studies have focused on specific patient populations, settings, or regions, limiting the generalizability of the findings to a broader range of COVID-19 patients, and e. the COVID-19 pandemic is still relatively recent, and many remote patient monitoring studies have focused on short-term outcomes; therefore, it has limited long-term data available on the effectiveness and sustainability of ICT-based remote monitoring for COVID-19 patients. Despite these limitations, this systematic literature review provides a valuable synthesis of the available evidence in ICT-based remote and automatic COVID-19 patient monitoring and care.

## Conclusion

This systematic literature review has revealed different e-Health methods, associated RMTs, approaches, different collected data types, adoption of ICT tools for automatic remote patient monitoring, benefits, and constraints for RMTs in COVID-19 case. Digital health tools can improve efficiency, accessibility, and quality of care. A wide range of wearable sensors has shown great potential for non-invasive, early, and on-time diagnosis and monitoring of COVID-19 patients. Advances in smart wearable sensors or devices represent an excellent opportunity to accelerate the transformation of connected medical technology.

RPM can be a good alternative for COVID-19 patient monitoring based on their health risks and needs. While improved home monitoring may enhance safety and value, empirical evidence to support the benefits of this approach is limited. Home monitoring and the home hospital model offer the potential to transform care and enable many hospital patients to receive care at home. However, healthcare systems must partner with technology producers to accelerate learning and create excellent value for patients, clinicians, and healthcare organizations. More research is required to understand what strategic features are helpful, their cost-effectiveness, and relevancy to different age groups. The choice of e-Health communication services plays a vital role in the success of remote disease management. Further research should focus on the security and privacy of COVID-19 monitoring systems that handle sensitive patient data.

AI-based disease management is likely to be highly controversial among regulators and healthcare professionals, depending on how much freedom AI is given to determine remote COVID-19 patient treatment. However, it also has the potential to free human doctors from routine disease management tasks, allowing them to focus on patients who do not respond to standard treatment options. It will enable doctors to treat many more patients than they currently have. Diagnosis requires a doctor; however, their treatment can be largely automated once a patient is diagnosed. As remote patient monitoring systems evolve into AI-driven disease management systems, we can radically improve the availability and quality of care and help address the healthcare cost crisis. Collaboration that leverages the expertise of clinicians, data scientists, engineers, and nurses will be critical to facilitate this progress and may be even more desirable if a further wave of the pandemic occurs.

### Supplementary Information

**Additional file 1. **We added a PRISMA 2009 Checklist document as a part of this systematic literature review.

**Additional file 2. **Adopted publication types, e-Health methods, RMTs, and corresponding collected data as a part of RPM in COVID-19 case.

**Additional file 3. **Adopted approaches to design and develop an RPM program.

**Additional file 4. **Studies on trials and corresponding outcomes with model performances (if applicable).

**Additional file 5. **Studies related to automatic RPM process.

## Data Availability

All data generated or analysed during this study are included in this published article [and its supplementary information files].

## Abbreviations

AI: Artificial Intelligence
ANN: Artificial Neural Network
CNN: Convolutional Neural Network
CART: Decision Tree Regression
BioMeTs: Biometric Monitoring Technologies
BLE: Bluetooth-Enabled
BR: Bayesian Ridge
CDD: COVID-19 Disease Diagnosis
AUC: Area Under the Curve
MSE: Mean Squared Error
MAE: Mean Absolute Error
DL: Deep Learning
eHealth: Electronic Health
GPS: Global Positioning System
EWS-2: Early Warning Score 2
ECG: Electrocardiogram
GA: Genetic Algorithm
HMP: Home Monitoring Program
ICT: Information and Communication Technology
IoT: Internet of Things
IoMT: Internet-of-Medical-Things
KNN: K-Nearest Neighbors
LSTM: Long-short-Term Memory
LR: Logistic Regression
LDA: Linear Discriminant Analysis
mHealth: Mobile Health
ML: Machine Learning
NB: Naive Bayesian
PRISMA: Preferred Reporting Items for Systematic Reviews and Meta-Analyses
RMT: Remote Monitoring Technology
RQ: Research Questions
RPM: Remote Patient Monitoring
RNN: Recurrent Neural Network
SANRA: Scale for the Assessment of Narrative Review Articles
SpO2: Oxygen Saturation
SVM: Support Vector Machine
UiA: University of Agder
