# Supplementary material for: A systematic review and knowledge mapping on ICT-based remote and automatic COVID-19 patient monitoring and care
Source: BMC Health Serv Res. 2023 Sep 30;23:1047. doi: 10.1186/s12913-023-10047-z (PMC10543863; doi:10.1186/s12913-023-10047-z)
Supplement: Supplementary file 3 — Additional file 3. Adopted approaches to design and develop an RPM program. [file 12913_2023_10047_MOESM3_ESM.docx]

**Supplementary Table 2.** Adopted approaches to design and develop an RPM program.

| Approaches | Studies* | Findings |
| --- | --- | --- |
| Home Monitoring Program (HMP) | [1] | HMP model of care may be implemented on a large scale to reduce the burden of COVID-19 on hospitals. A significant benefit of the HMP is the ability to reassure patients that if their condition worsens while in isolation outside the hospital, they will receive appropriate interventions. |
| Adapted staffing and billing models | [20] | Despite its long history, telemedicine is not used as the primary means of delivering pediatric cardiac care in most healthcare settings. Due to the COVID-19 pandemic, telemedicine in pediatric cardiology will impact our daily practice. This approach can be effective in telehealth-based RPM. |
| Classic tele-consultation model | [30] | The model can be useful in RPM for COVID-19 case to monitor mental health and consult accordingly. |
| Telemonitoring | [31] | Telemedicine and telemonitoring may improve patient autonomy to manage their diabetes in COVID-19 case. |
| Tele-COVID | [33][36] | Video calling in social media or dedicated apps, live video conferencing platforms, and personal webcams represent a valuable strategy for effective and real-time COVID-19 patient communication and healthcare delivery despite limitations, especially technology difficulties and low bandwidth. |
| COVID-19 disease diagnosis (CDD) | [40] | First, examined the patient's symptoms and predicted the likelihood of infection. Then, depending on the possibility of infection, the patient's lungs were diagnosed and confirmed by automated analysis of X-ray or computed tomography (CT) images to confirm the presence of infection. |
| National early warning score 2 (NEWS-2) | [44] | It is a reference method for detecting changes in a patient's clinical status by monitoring a symptom-based risk index. A score is generated based on the patient's respiratory rate, oxygen saturation, systolic blood pressure, heart rate, level of consciousness, body temperature, and dependence on supplemental oxygen. |
| IoT-Health Monitoring system (IoT-HMS) | [44] | It presents a comprehensive IoT-based conceptual architecture that addresses the critical requirements of scalability, interoperability, network dynamics, context awareness, reliability, and privacy related to remote health monitoring of COVID-19 patients in hospitals and at home. |
| IoT-HMS-Framework | [45] | The proposed framework consists of five central units that will be used to collect data from wearable devices, receive results reports from quarantine centers, analyze data through machine learning, connect with healthcare professionals, and integrate with cloud infrastructure |
| High-intensity RPM care model | [46] | It focuses on patients with complex care needs requiring daily monitoring with clinical-grade home equipment and centralized RPM support. It is designed to support patients in precarious health conditions or who may experience severe complications from treatment. |
| Remote biomedical signal monitoring framework | [49] | It combines IoT, 5G communication and AI techniques. IoT devices are used to collect biomedical signals at the sensory level. The biomedical signal is then transmitted through the 5G network to the cloud server, where the GRU-AE deep learning model is deployed for time-series biomedical signal analysis. |
| IoT-Remote Monitoring Framework | [50] | Components involved on the Internet of Things, such as sensors, actuators, microcontrollers, and cloud-enabled systems, support patients at home instead of frequent hospital visits. |
| Wearable IoTs and Geo-Fencing Based Framework | [51] | The system consists of four layers, each with a different function: wearable sensor layer, IoT gateway layer, cloud server layer, and client application layer for visualization and analysis. |
| Ontology-based IoT framework | [54] | The proposed model is an IoT-based remote access and alert bio-wearable sensor system for early detection of COVID-19 based on an ontology approach using one-dimensional sensing biomedical signals such as ECG, PPG, temperature, and accelerometer. |
| Design Science Approach | [56] | The digital platform was developed for the project's specific objectives and was successfully piloted in three primary health centers in the Lisbon Health District. Healthcare professionals were able to treat their first patients remotely safely and thoroughly and were very satisfied. |
| CovIoT model design | [61] | The system can be used as an end-to-end COVID patient monitoring system and for automated disinfection. |
| CovidDetNet model design | [63] | The proposed approach has effectively and efficiently identified and classified COVID-19 using chest X-rays. |
| SatNav E@syCare model design | [64] | The telemedicine platform enables remote monitoring, reduces contact between patients and doctors, and automates medical processes. |
| Vector Algorithm design | [65] | The Vector algorithm enables a new approach to the management of COVID-19 patients by early detection of interstitial pneumonia from lung sounds. |
| Applied image processing | [66] | The proposed model is promising, can quickly and accurately classify chest images, and helps doctors as the second reader in their final decision. |
| Smart COVID-Shield model design | [67] | It is more accurate and faster than heat gun detection. It automatically detects common symptoms such as fever and cough and ensures proper adherence to social distancing norms. |
| Federated MLaaS model design | [68] | The framework supports data preprocessing and feature selection to extract relevant indicators for COVID-19 disease classification. It can progressively learn and train over time on collected data as well as historically stored data. |
| CycleGAN-Inception model design | [70] | The CycleGAN algorithm has been proposed for data augmentation of COVID-19 X-ray images. A new method for identifying COVID-19 using X-ray images is proposed. |
| Bi-LSTM network on the deep features | [72] | The study proposes a deep learning-based method for detecting Covid-19 and undetected cases using chest X-rays. Despite the small number of Covid-19 images in the dataset, the Bi-LSTM network outperformed the deep-neural net (DNN) with an accuracy of 97.6%. |
| Metaheuristics Convolute Networks with IoT-Based Wearable Device | [73] | The system uses wearable IoT sensors and cloud and network layers to remotely check the health status of patients. Each layer has a specific function in the COVID-19 symptom monitoring process. The first layer collects patient health information and transmits it to the second layer, which stores this data in the cloud. The network examines health data and alerts patients, helping users take immediate action. Finally, the network layer notifies family members to take appropriate action. |
| e-CoVig model deisgn | [75] | The system has a mobile app, a web/cloud platform, and a cost-effective specific device for recording health parameters. The system architecture is flexible and can be configured for different operating conditions. It provides a range of capabilities to remotely report symptoms, vital signs, and other clinical information to health services caring for COVID-19 patients. Data captured in the mobile application is automatically sent to the web/cloud application and made available to medical staff in real-time. |
| Symptoms Forecasting and Disease Detection (SFDD) | [77] | Integrating autoregressive integral moving averages to design predictive models to find the kth prediction of patient-observed health symptoms and introducing selection based on Akaike's information criterion to see the current best-fit predictive model. |

* We found overlapping research topics in several studies.
