# Supplementary material for: A systematic review and knowledge mapping on ICT-based remote and automatic COVID-19 patient monitoring and care
Source: BMC Health Serv Res. 2023 Sep 30;23:1047. doi: 10.1186/s12913-023-10047-z (PMC10543863; doi:10.1186/s12913-023-10047-z)
Supplement: Supplementary file 4 — Additional file 4. Studies on trials and corresponding outcomes with model performances (if applicable). [file 12913_2023_10047_MOESM4_ESM.docx]

**Supplementary Table 3.** Studies on trials and corresponding outcomes with model performances (if applicable).

| Study | Study design | Group Size | Age group | Dataset used | Research findings | Model performance |
| --- | --- | --- | --- | --- | --- | --- |
| [1] | Random controlled study | 46 | - | - | HMP identifies clinical exacerbations. All participants were positive about HMP. | - |
| [5] | Random controlled study | 162 of 173 | median of 38, range 11-79 | - | Community-based virtual healthcare is safe to treat most COVID-19 patients and can be rapidly implemented in an urban pandemic management setting. | - |
| [22] | Random controlled study | 200-1000 | Age ≥18 | - | Insights for detecting the disease progression of COVID-19 by monitoring physiological data and possible ways to reduce disease transmission. | - |
| [36] | Random controlled study | 95 | Age: > 60 | - | Tele-vital signs monitoring system can be an intelligent solution to the COVID-19 crisis. | - |
| [38] | Empirical study | - | - | Novel Corona Virus 2019 Dataset in Kaggle | Data analysis showed a positive correlation between patient gender and death and showed that most patients were between 20 and 70 years. | Accuracy: 94%, F1-score: 86% |
| [40] | Empirical study | - | - | Symptom datasets and COVID-19 image datasets (CT-scan and X-ray) | A CDD scheme has been proposed. | Accuracy: 90% |
| [46] | Random controlled study | 8548 for RPM and 7074 for feasibility analysis | - | - | RPM technology engagement rate was 78.9%. | - |
| [47] | Random controlled study | 34 | median of 32 | - | Investigated the potential of wearable biosensors and machine learning-based analysis of physiological parameters to detect clinical exacerbations. | - |
| [49] | Random controlled study | 2000 | Mixed | - | This research essentially promotes the development of biomedical signal-monitoring technology in the remote monitoring of COVID-19 patients. | - |
| [55] | Random controlled study | 112 | Mean age 49 (SD:17.6) 60.7% female | - | The most reported comorbidities were hypertension (36.3%), hypercholesterolemia (26.5%), and diabetes (17.7%). The most reported symptoms were dyspnea (55.4%) and anxiety (55.4%). Anxiety was listed as the most severe symptom (9.8%). | - |
| [58] | Random controlled study | 162 | Mean age 38 years, range 11-79 years | - | Community-based virtual healthcare is safe for treating most COVID-19 patients and can be rapidly implemented in urban settings for pandemic management. | - |
| [61] | Random controlled study | 100 | - | - | Compared to industry standard equipment. These low error values ​​indicate the high accuracy of our proposed system. | MAE and MSE ​​were 0.79 and 1.03 for the oximeter, 1.22 and 0.70 for the heart rate monitor, and 1.07 and 1.28 for the temperature monitor |
| [65] | Random controlled study | 28 | - | - | Lung sounds data represent a reliable biomarker of COVID-19 pneumonia. | - |
| [66] | Empirical study | - | - | Cohen dataset and Kermany dataset | - | Accuracy: 98% |
| [70] | Empirical study | - | - | COVID-19-X-ray-and-CT-Chest-Images and Chest-Xray-Dataset | X-ray imaging is one of the most popular techniques for visualizing the effects of viruses on the lungs. | Accuracy: 94.2%, AUC: 92.2%, MSE: 0.27, and MAE: 0.16. |
| [74] | Empirical study | 956 | - | Lung CT images | Automatic classification of normal, mild, moderate, and severe COVID-19 based on pulmonary parenchymal involvement. | - |
| [78] | Mixed study (Random controlled and empirical) | 1208 | - | Chest X-rays of 396 COVID-19 patients | The proposed convolutional neural network-based algorithm was able to classify disease severity into four categories (normal, mild, moderate, and severe). | AUC: 93% |
| [79] | Empirical study | - | - | Covid-Chest Xray and Chex-Pert dataset | The proposed intelligent healthcare system integrates IoT-cloud technologies. | Accuracy: 98.6%, F1-score: 97.87% F1 |
