# Supplementary material for: A systematic review and knowledge mapping on ICT-based remote and automatic COVID-19 patient monitoring and care
Source: BMC Health Serv Res. 2023 Sep 30;23:1047. doi: 10.1186/s12913-023-10047-z (PMC10543863; doi:10.1186/s12913-023-10047-z)
Supplement: Supplementary file 5 — Additional file 5. Studies related to automatic RPM process. [file 12913_2023_10047_MOESM5_ESM.docx]

**Supplementary Table 4.** Studies related to automatic RPM process.

| Study | ICT tools | Target |
| --- | --- | --- |
| [42] | Deep Learning and IoT | Architecture to analyze cough and breathing acoustic characteristics. |
| [43] | Deep learning | Technologies based on standard machine learning, image and signal processing techniques can be easily deployed on smartphones for continuous vital sign monitoring. |
| [45] | Machine Learning | The predictive model is constructed based on documented cases in a public COVID-19 dataset, and manual feature extraction combined with patient medical history. |
| [47] | Machine Learning | Independent analysis of physiological parameters, symptoms and other medical data generated a machine learning-derived index that reflects overall health status, the Biological Vital Index. |
| [49] | Deep learning | AI, IoT, and related technologies have been applied in biomedical signal monitoring and remote health monitoring. |
| [60] | IoT | The study developed a simple, reliable, and low-cost microcontroller-based wireless vital sign monitoring system with high mobility and low power consumption. |
| [61] | IoT | This study developed a novel Arduino-based automatic hand sanitizer dispenser that integrates an oximeter, heart rate monitor, contactless body temperature sensor, and voice assistant feedback. |
| [62] | Deep learning, IoT | This study proposes a framework for the automatic segmentation of COVID Radiopedia and Medseg datasets using WoT and traditional U-Net with EfficientNet B0. |
| [63] | Deep learning | This study made a three-level classification (COVID-19, pneumonia, and normal) because its automatic prediction and detection can help doctors in timely treatment according to the cause of infection. |
| [64] | Deep learning | The performance of VECTOR was compared with diagnostic imaging modalities, namely lung ultrasound, chest X-ray, and high-resolution computed tomography, which were accepted as ground truth. |
| [66] | Deep learning | The model inputs chest x-rays and can extract x-ray patterns from chest x-rays to gain valuable information and monitor structural differences in the lungs caused by disease. |
| [67] | IoT | It consists of three modules, including a cough detection module (CDM), a temperature detection module (TDM), and a distance calculation module (DCM). The braces are equipped with a passive infrared (PIR) sensor and a temperature sensor to monitor persistent coughing patterns and high body temperature. An ultrasonic sensor scans 6 feet to track a person's social distancing norms. |
| [68] | Federated Machine Learning | The learning-based framework maintains an accurate decision model shared by the two joint streaming Machine Learnings or ML (i.e., the same learned model is inherited by the streaming ML residing in the cloud layer and the streaming ML installed in the fog layer). The proposed model ensures long-term decision-making using federated batch ML. |
| [69] | Deep learning | Acoustic analysis of breath sounds from COVID-19 patients revealed changes in frequency content. Deep learning classifiers, convolutional neural networks (CNNs), provide a promising diagnostic tool from the acoustic analysis. |
| [70-72][76][78][79] | Deep learning | The models are capable of automatic visualizing the effects of viruses on the lungs from X-ray imaging. Deep learning-based methods are helpful for the automatic detection of COVID-19/coronavirus patients from X-ray and CT images. |
| [74] | Machine learning | This proposed model may work well and be used as a diagnostic tool to quantify lung involvement in COVID-19 to monitor disease progression. |
| [77] | Machine learning | In the proposed model, features are extracted from the forecasts of each symptom, patterns are found for each patient, and patterns are learned through the K-Means algorithm to identify symptomatic and asymptomatic patients intelligently. |
| [80] | Deep learning, IoT | This study monitors COVID-19 patients using deep learning using a 5G-enabled automated real-time cardiovascular monitoring system with open-source frameworks like Kafka, Flink, and TensorFlow to realize personal data transmission and monitoring model construction. |
| [81] | Machine learning | This study proposes a hybrid framework for efficient and automatic COVID-19 detection and diagnosis from cough audio signals using different ML algorithms. The framework's accuracy has been improved by using genetic algorithms and ML techniques. |
